# Supplementary material for: A UMLS-based spell checker for natural language processing in vaccine safety
Source: BMC Med Inform Decis Mak. 2007 Feb 12;7:3. doi: 10.1186/1472-6947-7-3 (PMC1805499; doi:10.1186/1472-6947-7-3)
Supplement: Additional file 13 — RAP application directory. Sets up directory for RAP (RDF application for PHP) source code [file 1472-6947-7-3-S13.gz › rap/doc/phpdoc/default/DC.html]

xml version="1.0" encoding="iso-8859-1"?


Docs For Class DC


## Class DC

Description

Description |
Methods (details)

Located in /vocabulary/DC\_C.php (line 24)

Method Summary

Description |
Methods (details)

void
ABSTRACT\_
()

void
ACCESS\_RIGHTS
()

void
ALTERNATIVE
()

void
AUDIENCE
()

void
AVAILABLE
()

void
BIBLIOGRAPHIC\_CITATION
()

void
BOX
()

void
COLLECTION
()

void
CONFORMS\_TO
()

void
CONTRIBUTOR
()

void
COVERAGE
()

void
CREATED
()

void
CREATOR
()

void
DATASET
()

void
DATE
()

void
DATE\_ACCEPTED
()

void
DATE\_COPYRIGHTED
()

void
DATE\_SUBMITTED
()

void
DCMI\_TYPE
()

void
DESCRIPTION
()

void
EDUCATION\_LEVEL
()

void
EVENT
()

void
EXTENT
()

void
FORMAT
()

void
HAS\_FORMAT
()

void
HAS\_PART
()

void
HAS\_VERSION
()

void
IDENTIFIER
()

void
IMAGE
()

void
IMT
()

void
INTERACTIVERESOURCE
()

void
ISO639\_2
()

void
ISO3166
()

void
ISSUED
()

void
IS\_FORMAT\_OF
()

void
IS\_PART\_OF
()

void
IS\_REFERENCED\_BY
()

void
IS\_REPLACED\_BY
()

void
IS\_REQUIRED\_BY
()

void
IS\_VERSION\_OF
()

void
LANGUAGE
()

void
LCC
()

void
LCSH
()

void
LICENSE
()

void
MEDIATOR
()

void
MEDIUM
()

void
MESH
()

void
MODIFIED
()

void
MOVINGIMAGE
()

void
PERIOD
()

void
PHYSICALOBJECT
()

void
POINT
()

void
PUBLISHER
()

void
REFERENCES
()

void
REPLACES
()

void
REQUIRES
()

void
RFC1766
()

void
RFC3066
()

void
RIGHTS
()

void
RIGHTS\_HOLDER
()

void
SERVICE
()

void
SOFTWARE
()

void
SOUND
()

void
SOURCE
()

void
SPATIAL
()

void
STILLIMAGE
()

void
SUBJECT
()

void
TABLE\_OF\_CONTENTS
()

void
TEMPORAL
()

void
TEXT
()

void
TGN
()

void
TITLE
()

void
TYPE
()

void
UDC
()

void
URI
()

void
VALID
()

void
W3CDTF
()

Methods

Description |
Methods (details)

ABSTRACT\_ (line 111)

void

ABSTRACT\_
()

ACCESS\_RIGHTS (line 117)

void

ACCESS\_RIGHTS
()

ALTERNATIVE (line 123)

void

ALTERNATIVE
()

AUDIENCE (line 129)

void

AUDIENCE
()

AVAILABLE (line 135)

void

AVAILABLE
()

BIBLIOGRAPHIC\_CITATION (line 141)

void

BIBLIOGRAPHIC\_CITATION
()

BOX (line 323)

void

BOX
()

COLLECTION (line 421)

void

COLLECTION
()

CONFORMS\_TO (line 147)

void

CONFORMS\_TO
()

CONTRIBUTOR (line 26)

void

CONTRIBUTOR
()

COVERAGE (line 32)

void

COVERAGE
()

CREATED (line 153)

void

CREATED
()

CREATOR (line 38)

void

CREATOR
()

DATASET (line 427)

void

DATASET
()

DATE (line 44)

void

DATE
()

DATE\_ACCEPTED (line 159)

void

DATE\_ACCEPTED
()

DATE\_COPYRIGHTED (line 165)

void

DATE\_COPYRIGHTED
()

DATE\_SUBMITTED (line 171)

void

DATE\_SUBMITTED
()

DCMI\_TYPE (line 329)

void

DCMI\_TYPE
()

DESCRIPTION (line 50)

void

DESCRIPTION
()

EDUCATION\_LEVEL (line 177)

void

EDUCATION\_LEVEL
()

EVENT (line 433)

void

EVENT
()

EXTENT (line 183)

void

EXTENT
()

FORMAT (line 56)

void

FORMAT
()

HAS\_FORMAT (line 189)

void

HAS\_FORMAT
()

HAS\_PART (line 195)

void

HAS\_PART
()

HAS\_VERSION (line 201)

void

HAS\_VERSION
()

IDENTIFIER (line 62)

void

IDENTIFIER
()

IMAGE (line 439)

void

IMAGE
()

IMT (line 335)

void

IMT
()

INTERACTIVERESOURCE (line 445)

void

INTERACTIVERESOURCE
()

ISO639\_2 (line 347)

void

ISO639\_2
()

ISO3166 (line 341)

void

ISO3166
()

ISSUED (line 237)

void

ISSUED
()

IS\_FORMAT\_OF (line 207)

void

IS\_FORMAT\_OF
()

IS\_PART\_OF (line 213)

void

IS\_PART\_OF
()

IS\_REFERENCED\_BY (line 219)

void

IS\_REFERENCED\_BY
()

IS\_REPLACED\_BY (line 225)

void

IS\_REPLACED\_BY
()

IS\_REQUIRED\_BY (line 231)

void

IS\_REQUIRED\_BY
()

IS\_VERSION\_OF (line 243)

void

IS\_VERSION\_OF
()

LANGUAGE (line 68)

void

LANGUAGE
()

LCC (line 353)

void

LCC
()

LCSH (line 359)

void

LCSH
()

LICENSE (line 249)

void

LICENSE
()

MEDIATOR (line 255)

void

MEDIATOR
()

MEDIUM (line 261)

void

MEDIUM
()

MESH (line 365)

void

MESH
()

MODIFIED (line 267)

void

MODIFIED
()

MOVINGIMAGE (line 451)

void

MOVINGIMAGE
()

PERIOD (line 371)

void

PERIOD
()

PHYSICALOBJECT (line 457)

void

PHYSICALOBJECT
()

POINT (line 377)

void

POINT
()

PUBLISHER (line 74)

void

PUBLISHER
()

REFERENCES (line 273)

void

REFERENCES
()

REPLACES (line 279)

void

REPLACES
()

REQUIRES (line 285)

void

REQUIRES
()

RFC1766 (line 383)

void

RFC1766
()

RFC3066 (line 389)

void

RFC3066
()

RIGHTS (line 80)

void

RIGHTS
()

RIGHTS\_HOLDER (line 291)

void

RIGHTS\_HOLDER
()

SERVICE (line 463)

void

SERVICE
()

SOFTWARE (line 469)

void

SOFTWARE
()

SOUND (line 475)

void

SOUND
()

SOURCE (line 86)

void

SOURCE
()

SPATIAL (line 297)

void

SPATIAL
()

STILLIMAGE (line 481)

void

STILLIMAGE
()

SUBJECT (line 92)

void

SUBJECT
()

TABLE\_OF\_CONTENTS (line 303)

void

TABLE\_OF\_CONTENTS
()

TEMPORAL (line 309)

void

TEMPORAL
()

TEXT (line 487)

void

TEXT
()

TGN (line 395)

void

TGN
()

TITLE (line 98)

void

TITLE
()

TYPE (line 104)

void

TYPE
()

UDC (line 401)

void

UDC
()

URI (line 407)

void

URI
()

VALID (line 315)

void

VALID
()

W3CDTF (line 413)

void

W3CDTF
()

Documentation generated on Fri, 17 Dec 2004 16:14:44 +0100 by phpDocumentor 1.3.0RC3
